# Multiplex polymerase chain reaction detection of enteropathogens in sewage in Norway

**DOI:** 10.1186/s13028-019-0445-5

**Published:** 2019-03-04

**Authors:** Øyvind Ørmen, Kristian Aalberg, Elisabeth Henie Madslien

**Affiliations:** 1 Department of Veterinary Services and Force Health Protection, Norwegian Armed Forces Joint Medical Services, Postmottak, PO Box 800, 2617 Lillehammer, Norway; 2 Comprehensive Defence Division, Norwegian Defence Research Establishment, PO Box 25, 2027 Kjeller, Norway

**Keywords:** Enteropathogens, Filmarray, PCR, Sewage, Wastewater treatment plant

## Abstract

The primary objective of this small-scale study was to investigate the occurrence of enteropathogens in sewage (municipal wastewater) in Norway using the commercially available FilmArray^®^ multiplex polymerase chain reaction (PCR) system with the gastrointestinal (GI) panel. Our findings indicate that DNA/RNA of several enteropathogens are present simultaneously in Norwegian wastewater systems. The spectre was broad even in smaller communities. With some exceptions, occurrence corresponded more or less to the reported cases of infectious human gastrointestinal disease in the same geographical regions. The effects of different sewage purification techniques were assessed on a limited number of samples indicating that neither chemical nor biological treatment was sufficiently effective to reduce gene material from the pathogens to undetectable levels. Further studies are required to assess the performance and suitability FilmArray^®^ multiplex PCR when used on collective sewage samples in outbreak situations. Additionally, screening sewage samples using multiplex-PCR could be valuable in order to detect new and emerging pathogens and for preliminary analysis of samples before proceeding to more work demanding confirmatory techniques.

## Findings

Detection and identification of enteropathogens in environmental samples have traditionally relied on a combination of different diagnostic tools, including microscopy, enrichment, culture, antigen–antibody-interaction based tests and polymerase chain reaction (PCR) based molecular techniques. Multiplex PCR assays permit rapid detection of nucleic acids from multiple infectious agents, including bacteria, viruses and parasites and analysis of sewage (municipal wastewater) to provide early warning of foodborne outbreaks was previously suggested [1]. This study applied the commercially available FilmArray^®^ multiplex PCR system (BioFire-bioMérieux, France) to explore the occurrence of enteropathogens in municipal wastewater, in the south eastern part of Norway. The FilmArray^®^ Gastrointestinal (GI) panel is US Food and Drug Administration (FDA)-approved for diagnostic purposes on clinical faecal samples. However, limited data are available regarding its performance on environmental samples [2].

In this study, occurrence of pathogen specific molecular markers (DNA/RNA) was compared to the reported prevalence of the corresponding diseases in humans in the corresponding region, the sewage purification techniques and the size of the populations.

Sampling was conducted during winter/spring/early summer 2018 at various municipal wastewater treatment facilities (n = 11), located along the course of the river Glomma. One litre of untreated and treated wastewater was collected in sterile bottles from each treatment plant. Samples were prepared by filtrating 900 mL of sampled water through Sleicher & Schull 597 filter paper under vacuum assistance. Filters from each sample were subsequently homogenized in the remaining 100 mL of sample using a stomacher 400 Seward. FilmArray^®^ reagent pouches were loaded with 500 µL of homogenated sample, 1.5 mL hydrating solution FilmArray^®^ Injection Vial-GI before analysis by FilmArray^®^ system using the gastrointestinal (GI) panel targeting 22 different pathogens.

Analysis identified molecular markers of different *Escherichia coli* pathotypes, *Yersinia enterocolitica* and *Campylobacter* spp. indicating that these are the most prevalent bacterial pathogens in untreated sewage (Table 1). Enteroaggregative *E. coli* (EAEC) and *Y. enterocolitica* were detected at all sampling sites, while *Campylobacter* spp. was detected at 8 out of 11 sampling sites. These findings are partially compatible with reported human cases of gastrointestinal infections in the same geographical area and period [1]. According to the Norwegian Surveillance System for Communicable Diseases (MSIS), the most frequently diagnosed enteropathogens in the same municipalities are in descending order: *Campylobacter* spp., *Salmonella enterica* sp. *enterica* biovars, and various *E. coli* pathotypes [3]. Our frequent detection of *Y. enterocolitica* does not match the single reported case of clinical disease in the region, which could be due to a number of reasons such as underreporting, subclinical carriers, low target specificity (pathogenic vs. non-pathogenic strains), environmental/animal sources and the ability of this agent to grow and survive in wastewater systems. Earlier studies utilizing traditional culture-based methods have demonstrated *Yersinia* spp. occurrence in 54% to 90% of sewage samples, where a majority of isolates have been non-pathogenic to humans [4, 5]. *Clostridium difficile* was detected at one site, while according to surveillance data constituting one of the most frequently diagnosed agents of gastrointestinal disease. Infection by *C. difficile* is typically acquired by hospitalized patients undergoing antibacterial therapy; this may account for the relatively high number of reported cases [6]. *Salmonella* spp. were detected at a moderate number of sites, confirming the low prevalent *Salmonella* status in Norway compared to other European countries, where *Salmonella* spp. have been shown to occur more frequently in sewage [7].

**Table 1:** Enteropathogens detected in untreated sewage in Norway, compared to diagnosed cases of disease in corresponding municipalities

Agent	Number of positive plants (n = 11)	Number of patients reported in January–June 2018
*Campylobacter* spp.	8	86
*Clostridium difficile* toxin A/B	1	55
*Plesiomonas shigelloides*	4	No data
*Salmonella* spp.	3	33
*Vibrio* spp.	2	No data
*Vibrio cholerae*	1	0
*Yersinia enterocolitica*	11	1
Enteroaggregative *E. coli*	11	10
Enteropathogenic *E. coli*	7	
Enterotoxigenic *E. coli*	7	
Shigatoxin positive *E. coli*	9	10
*E. coli* O 157	1	
*Shigella*/Enteroinvasive *E. coli*	4	3
Adenovirus F40/41	11	204^a^
Astrovirus	2	18^a^
Norovirus GI/GII	6	290^a^
Rotavirus A	7	37^a^
Sapovirus (I, II, IV and V)	6	66^a^
*Cryptosporidium* spp.	1	9
*Cyclospora cayetanensis*	0	0
*Entamoeba histolytica*	1	0
*Giardia lamblia*	10	7

^a^Virus data contains positive samples from the medical laboratories serving the eastern part of Norway and not restricted to the municipal producing the wastewater

Molecular markers of all five viral agents included in the FilmArray ^®^ GI-panel were detected by the screening of untreated sewage. Adenovirus was detected at every sampled site, which coincides with its frequent isolation at regional medical diagnostic laboratories, and earlier publications describing Adenovirus as the enteric virus most commonly isolated from wastewater [8, 9]. Rotavirus was detected at 7 out of 11 sampled sites, despite constituting only a small share of diagnosed cases of viral diarrheal disease in the region. Rotavirus infection in adults typically proceeds asymptomatically or with mild clinical signs, influencing the degree to which cases are reported [10]. Norovirus and Sapovirus were both detected at 6 sites. Sapoviruses are generally considered less pathogenic than Norovirus, which may explain its frequent isolation in sewage compared to its reduced clinical relevance (Table 1).

Sample analysis demonstrated *Giardia lamblia* as the most frequent protozoan agent found in untreated sewage. The parasite was detected in 9 out of 11 sites. *Cryptosporidium* spp. and *Entamoeba* spp. were both isolated once each, while traces of genetic material from *Cyclospora cayetanensis* was not found. Detection of *Giardia* spp. in the absence of *Cryptosporidium* spp. at a majority of test sites was an unexpected finding, and is neither consistent with MSIS clinical data nor earlier surveys of sewage parasite occurrence [11]. According to disease surveillance, *Entamoeba histolytica* is a sporadic finding, and *Cyclospora cayetanensis* has not yet been isolated in Norway [3], both of which is in accordance with our findings.

The samples were collected from sewage treatment plants of which different purification techniques were used (Table 2). We collected samples pre and post purification to evaluate any differences in detection frequency as a result of the treatment process. Mechanical treatment, comprising filtration or sieving, had no reducing effect on the number of pathogen species detected. The increase of pathogens after mechanical treatment could be explained by the addition of septic waste after the filtration. Chemical treatment with flocculation and sedimentation resulted in a 50% reduction in the number of identified pathogens, this is in accordance with the reduction in coliform bacteria reported by Curtis [12]. Further reduction was observed subsequent to a final biological purification step. However, pathogens were still detected in several of the samples after the chemical and biological treatment steps. Whether this represent viable cells or nucleic material from dead cells only remains to be elucidated. Nevertheless, our results indicate that sewage might be a source of environmental spread of genetic material from enteropathogens in Norway.

**Table 2:** Occurrence of enteropathogens in untreated and treated wastewater; number of detections grouped according to type of purification system

Agent	Mechanical purification (n = 2)		Mechanical, and chemical purification (n = 6)		Mechanical, chemical and biological purification (n = 3)	
	Untreated	Treated	Untreated	Treated	Untreated	Treated
*Campylobacter* spp.	0	1	4	1	3	0
*Clostridium difficile* toxin A/B	0	0	1	0	0	0
*Plesiomonas shigelloides*	0	0	2	0	2	0
*Salmonella* spp.	0	1	2	0	0	0
*Vibrio* spp.	0	0	2	0	0	0
*Vibrio cholerae*	0	0	1	0	0	0
*Yersinia enterocolitica*	2	2	6	6	3	2
Enteroaggregative *E. coli*	2	2	6	4	3	2
Enteropathogenic *E. coli*	1	1	1	4	0	2
Enterotoxigenic *E. coli*	0	1	4	2	2	0
Shigatoxin positive *E. coli*	1	1	5	1	3	0
*E. coli* O 157	0	0	0	1	1	0
*Shigella*/Enteroinvasive E*. coli* coli	1	1	2	1	1	0
Positive samples bacteria	5	10	37	20	15	6
Reduction			46%		60%	
Adenovirus F40/41	2	2	6	3	3	1
Astrovirus	0	0	2	0	1	0
Norovirus GI/GII	0	0	2	2	5	1
Rotavirus A	0	0	2	0	5	3
Sapovirus (I, II, IV and V)	1	1	2	1	1	1
Positive samples virus	3	3	14	6	14	5
Reduction			57%		64%	
*Cryptosporidium* spp.	0	0	0	0	1	0
*Cyclospora cayetanensis*	0	0	0	0	0	0
*Entamoeba histolytica*	0	0	0	0	1	0
*Giardia lamblia*	1	1	6	2	3	1
Positive samples protozoa	1	1	6	2	5	1
Reduction			66%		80%	
Total positive samples	9	14	57	28	34	12

Grouping sampled treatment facilities according to population size, while removing atypical plants or plants serving atypical populations (1 plant removed due to recent direct septical sludge disposal into the system, 1 plant removed due to a large proportion of temporary inhabitants), permit the comparison of the pathogen profile of untreated sewage from different sized populations (Table 3). Molecular markers of a wide range of pathogens were present even in smaller wastewater systems without any reported ongoing local outbreaks. These findings highlight the importance of carriers and asymptomatic shedders in disease transmission, persistence of genetic material and possibly the ability for some enteropathogens to survive and grow in wastewater systems [13, 14]. It is also important to emphasize that most of the pathogens included in the panel are zoonotic. The rodent populations in the sewage systems, animal droppings from the surface run-off in the combined sewer and food industry (animal products) may have contributed to the findings.

**Table 3:** Occurrence of enteropathogens in untreated sewage; number of positive samples grouped according to treatment facility size (n = number of plants sampled)

Agent	2000–10,000 inhabitants (n = 3)	10,000–25,000 inhabitants (n = 3)	> 25,000 inhabitants (n = 3)
*Campylobacter* spp.	2	3	2
*Clostridium difficile* toxin A/B	1	0	0
*Plesiomonas shigelloides*	1	1	2
*Salmonella* spp.	1	1	0
*Vibrio* spp.	0	1	1
*Vibrio cholerae*	0	1	0
*Yersinia enterocolitica*	3	3	3
Enteroaggregative *E. coli*	3	3	3
Enteropathogenic *E. coli*	3	2	1
Enterotoxigenic *E. coli*	1	2	3
Shigatoxin positive *E. coli*	2	3	3
*E. coli* O 157	0	1	0
*Shigella*/Enteroinvasive *E. coli colicoli*	0	1	2
Adenovirus F40/41	3	3	3
Astrovirus	0	0	2
Norovirus GI/GII	1	3	2
Rotavirus A	2	2	3
Sapovirus	1	1	3
*Cryptosporidium* spp.	0	1	0
*Cyclospora cayetanensis*	0	0	0
*Entamoeba histolytica*	0	0	1
*Giardia lamblia*	3	3	3
Total	28	36	37

The prevalence of molecular markers of enteropathogens, as determined by the FilmArray^®^ GI system, is partially compatible with reported cases of gastrointestinal infections in the corresponding geographical areas. This indicates the applicability of multiplex PCR on sewage samples for the rapid culture-independent pathogen detection as an early warning in outbreak situations, particularly in confined areas such as military camps or hospitals when baseline levels are known. However, several factors might limit and challenge the use of this tool in outbreak investigations such as lack of proper baseline measures, inability to differentiate between dead and live cells, persistence of agents/nucleic acids in the sewage system, asymptomatic shedders and the risk of false positives/negatives in complex sewage samples. As such, these issues should be addressed in follow-on studies to guide the interpretation of this tool when used in outbreak investigations. Nevertheless, the FilmArray^®^ GI system seems to be a promising tool for the preliminary rapid screening of sewage samples before proceeding to more work demanding confirmatory analysis techniques.

## References

[1] Hellmér M, Paxéus N, Magnius L, Enache L, Arnholm B, Johansson A (2014). Detection of pathogenic viruses in sewage provided early warnings of hepatitis A virus and norovirus outbreaks. Appl Environ Microbiol..

[2] Gyawali P, Croucher D, Hewitt J (2018). Preliminary evaluation of BioFire FilmArray® Gastrointestinal Panel for the detection of noroviruses and other enteric viruses from wastewater and shellfish. Environ Sci Pollut Res..

[3] The Norwegian Institute of Public Health [Folkehelseinstituttet]. http://www.msis.no. Accessed 1 July 2018.

[4] Ziegert E, Diesterweg I (1990). The occurrence of *Yersinia enterocolitica* in sewage Untersuchungen zum Vorkommen *Yersinia enterocolitica* im Abwasser. Zentralbl Mikrobiol..

[5] Langeland G (1983). *Yersinia enterocolitica* and *Yersinia enterocolitica*-like bacteria in drinking water and sewage sludge. Acta Pathol Microbiol Immunol Scand B.

[6] Bouza E (2012). Consequences of *Clostridium difficile* infection: understanding the healthcare burden. Clin Microbiol Infect.

[7] Jones PW, Rennison LM, Lewin VH, Redhead DL (1980). The occurrence and significance to animal health of salmonellas in sewage and sewage sludges. J Hyg (Lond)..

[8] The Norwegian Institute of Public Health [Folkehelseinstituttet]. http://lab.fhi.no. Accessed 1 July 2018.

[9] Grondahl-Rosado RC, Yarovitsyna E, Trettenes E, Myrmel M, Robertson LJ (2014). A one-year study on the concentrations of norovirus and enteric adenoviruses in wastewater and a surface drinking water source in Norway. Food Environ Virol..

[10] Anderson EJ, Weber SG (2004). Rotavirus infection in adults. Lancet Infect Dis..

[11] Robertson LJ, Hermansen L, Gjerde BK (2006). Occurrence of *Cryptosporidium* oocysts and *Giardia* cysts in sewage in Norway. Appl Environ Microbiol..

[12] Curtis T, Mara D, Horan N (2003). Bacterial pathogen removal in wastewater treatment plants. Water and wastewater microbiology.

[13] Dumontet S, Scopa A, Kerje S, Krovacek K (2001). The importance of pathogenic organisms in sewage and sewage sludge. J Air Waste Manag Assoc..

[14] Ottoson J. Comparative analysis of pathogen occurrence in wastewater strategies or barrier function and microbial control; 2005. TRITA-LWR PhD Thesis: 1021 ISSN 1650-860.

